# Supplementary material for: Load forecasting method based on CEEMDAN and TCN-LSTM
Source: PLoS One. 2024 Jul 5;19(7):e0300496. doi: 10.1371/journal.pone.0300496 (PMC11226047; doi:10.1371/journal.pone.0300496)
Supplement: Attachment — Submitted filename: Response to Reviewers.docx [file pone.0300496.s002.docx]

Dear reviewers，

Thank you for reviewing my submitted thesis. After reading the review comments that you have made，I have revised my thesis in response to the comments you have made. The specific modifications are listed below:

First，I have uploaded the datasets used in this experiment and thesis for reference.

Secondly，I've made changes to the line spacing in the body text and the placement of the charts.

Thirdly, the citation for reference 20 in the text has been changed to the author's name.

Fourthly, the graphs of the LSTM in the text have been modified to those provided in the Appendix.

I have made changes in response to the review comments and I will resubmit the revised paper. Thank you again for your contribution to the review of my thesis.

Your sincerely,

Luo Heng

Cheng Hao

Liu Chen Nan

Suzhou , China
