# Supplementary material for: Load forecasting method based on CEEMDAN and TCN-LSTM
Source: PLoS One. 2024 Jul 5;19(7):e0300496. doi: 10.1371/journal.pone.0300496 (PMC11226047; doi:10.1371/journal.pone.0300496)
Supplement: Attachment — Submitted filename: Response to Reviewers.docx [file pone.0300496.s003.docx]

Dear reviewers，

Thank you for reviewing my submitted thesis. After reading the review comments that you have made，I have revised my thesis in response to the comments you have made. The specific modifications are listed below:

First,I have checked the journal's template for papers and changed all the first-level headings in the paper to bold, 18-point font, and the second-level headings in the paper to bold, 16-point font.

Secondly, The font size of the text has been changed to a uniform 12-point font size.

Thirdly ,I've changed the spacing of the text and the placement of the charts again.

Finally, All references cited in the main text have been changed to superscript citations.

I have made changes in response to the review comments and I will resubmit the revised paper. Thank you again for your contribution to the review of my thesis.

Your sincerely,

Luo Heng

Cheng Hao

Liu Chen Nan

Suzhou , China
